# Power analysis for random‐effects meta‐analysis

**DOI:** 10.1002/jrsm.1240

**Published:** 2017-04-04

**Authors:** Dan Jackson, Rebecca Turner

**Affiliations:** ^1^ MRC Biostatistics Unit Cambridge UK

**Keywords:** cochrane, empirical evaluation, random‐effects meta‐analysis, power calculations

## Abstract

One of the reasons for the popularity of meta‐analysis is the notion that these analyses will possess more power to detect effects than individual studies. This is inevitably the case under a fixed‐effect model. However, the inclusion of the between‐study variance in the random‐effects model, and the need to estimate this parameter, can have unfortunate implications for this power. We develop methods for assessing the power of random‐effects meta‐analyses, and the average power of the individual studies that contribute to meta‐analyses, so that these powers can be compared. In addition to deriving new analytical results and methods, we apply our methods to 1991 meta‐analyses taken from the Cochrane Database of Systematic Reviews to retrospectively calculate their powers. We find that, in practice, 5 or more studies are needed to reasonably consistently achieve powers from random‐effects meta‐analyses that are greater than the studies that contribute to them. Not only is statistical inference under the random‐effects model challenging when there are very few studies but also less worthwhile in such cases. The assumption that meta‐analysis will result in an increase in power is challenged by our findings.

## 1 INTRODUCTION

Meta‐analysis is now a very commonly used statistical tool. There are many motivations for including meta‐analyses in systematic reviews. The Cochrane Handbook^1^ (their section 9.1.3) gives 4 reasons for doing so: (1) to increase power, (2) to improve precision, (3) to answer questions not posed by the individual studies, and (4) to settle controversies or generate new hypotheses. The focus here is on the first of these reasons, namely, to increase power. To quote directly from the Cochrane Handbook, “Many individual studies are too small to detect small effects, but when several are combined there is a higher chance of detecting an effect.” This statement furthers our belief that there is a commonly held implicit assumption that meta‐analysis necessarily provides a way to increase statistical power and so detect effects of interest. In this paper, we will challenge this assumption. This issue should be of interest to applied analysts regardless of their preferences concerning whether to present confidence intervals or results from hypothesis tests. The former is often encouraged, and we would align ourselves with those who emphasise estimation over testing. Due to the connection between hypothesis testing and confidence intervals however, a significant hypothesis test result is equivalent to a confidence interval that excludes the null. All our results that follow are therefore immediately applicable to those who prefer to present confidence intervals because they can frame the findings of whether or not confidence intervals contain the null, and so whether they are able to detect an effect.

The power of a hypothesis test is the probability that the null hypothesis is rejected when it is false. Bayesian methods would be needed to instead calculate the probability that the null hypothesis is false, but here we focus on classical methods. Power analysis for meta‐analysis is a sufficiently important topic to warrant an entire chapter devoted to it in the introductory text by Borenstein et al^2^ (their chapter 29). The methods for power analysis that we develop below are of this conventional type and do not attempt to allow for multiple testing within the same review^3^ or sequential testing in updated reviews.^4^ We will compare the power of the individual studies and the power of the meta‐analysis to which they contribute, because both types of power are important considerations, which impact on each other in practice.^5^, ^6^ The planning of future studies may be based on the power or precision of meta‐analysis results.^5^, ^7^, ^8^

Our other main focus is on the use of random‐effects meta‐analyses. The random‐effects model^9^, ^10^, ^11^, ^12^ relaxes the strong, and usually difficult to defend, assumption made by the fixed‐effect model that the studies estimate the same true effect. The random‐effects model introduces two reasons for doubting that the resulting meta‐analyses will possess more power than the individual studies, both of which are directly related to the between‐study variance. The first reason is that, compared to the fixed‐effect model, the need to estimate an additional parameter in random‐effects meta‐analyses could result in power loss even when there is no between‐study heterogeneity; in general, power is lost when introducing further parameters to a statistical model. However, a more obvious concern is that if the between‐study heterogeneity is very large then the variation in the study estimates could be very considerable; by (albeit, correctly) allowing for this variation, the standard error of the pooled estimate could then be very large. If this occurs, then the corresponding hypothesis test will possess very little power.

Others have previously discussed power calculations under the random‐effects model.^13^, ^14^ Hedges and Pigott (2001)^15^ also discuss how the unknown between‐study variance complicates power analysis in the random‐effects setting. Although these methods are directly related to our new methods, and will also be used below, a key distinction between our new methods and most previous work is that we develop methods for evaluating the power whilst taking into account the uncertainty in the estimated between‐study variance.

We should however be clear from the outset that meta‐analysis and study specific hypothesis tests involve testing different types of hypotheses: in a meta‐analysis, we test whether or not the average effect is a particular value (for example zero), and for individual studies we test whether or not the true study specific treatment effect is a particular value. The distinction between these 2 types of hypotheses is especially clear when using the random‐effects model, where we assume that the true treatment effects differ across studies. Whilst recognising that the meta‐analysis and study specific hypothesis tests differ in this way, we will still compare the power of these two types of tests. We should also recognise that the study‐specific and fixed‐effect model hypothesis tests possess, under the assumptions that these methods make, the correct significance level. However, the conventional random‐effects model hypothesis test only retains the nominal significance level approximately. To more fairly compare the power of different hypothesis tests, we should use the same actual (and not just the nominal) significance level throughout, and this is only approximately the case here. Some of the power of the conventional random‐effects model hypothesis test is therefore an artifact of the approximate nature of the methods used.

One motivation for developing methods for power analysis under the random‐effects model is so that we can retrospectively determine the powers of the random‐effects meta‐analyses from a large sample of meta‐analyses from Cochrane. We therefore calculate what is sometimes referred to as the “observed power” in this empirical investigation. Then, by comparing the power of these meta‐analyses to the average power of the studies that contribute to them, we can empirically investigate the validity of the notion that random‐effects meta‐analyses result in an increase in power. This empirical investigation of a large number of random‐effects meta‐analyses is one important contribution of this paper. The new Monte Carlo method that we develop for this purpose is another important contribution. Retrospective power calculations have some value in practice because they have the potential to explain why effects were not detected. For example, a systematic reviewer might be disappointed or surprised not to detect a particular effect, but this is likely to be mitigated or explained by a calculation that reveals that the power was in any case very low. Although low power also manifests itself as wide confidence intervals, a power calculation provides a much more direct statement about the difficulty in detecting effects than a confidence interval. However this should not be taken to suggest that the usual statistical inferences, made for example by using confidence intervals, are in any sense deficient because they do not involve power calculations. Readers may also be able to suggest other reasons why retrospective power calculations could be of interest.

Despite this, we would not advocate the routine use of retrospective power calculations in meta‐analysis, rather they are likely to be useful in some instances to clearly convey the difficulty in detecting particular effects. In our empirical investigation, we retrospectively investigate the powers of random‐effects meta‐analyses so that these powers can be compared to study specific powers. Our retrospective power calculations are performed to answer the more general question of whether or not random‐effects meta‐analyses usually provide an increase in power, rather than to retrospectively investigate the power for any specific meta‐analysis. Those who might advocate the routine use of retrospective power calculations should examine the arguments made by Hoenig and Heisey^16^ and, in our opinion, consider amending their position.

Another motivation is to develop a method for power analysis that is most suitable for those who wish to perform such an analysis at the planning stage. As Borenstein et al^2^ point out, ideally “a power analysis should be performed when the review is being planned, and not after the review has been completed.” However, determining suitable information for a power calculation at the planning stage of a meta‐analysis or systematic review is challenging, because the power depends on quantities like the number and size of studies, which the systematic reviewer cannot directly control (although as Borenstein et al point out, we could modify the inclusion criterion to include more or fewer studies). We therefore develop a method that facilitates a meaningful power calculation for those planning reviews and that also allows for the uncertainty in the estimation of the between‐study variance. This is another important contribution of this paper. Power calculations are especially useful at this early stage, because they can be used to inform systematic reviewers about important practical issues such as whether they should modify the inclusion criterion to accommodate more studies, or wait until more studies are available, and so on. We strongly encourage power calculations at the planning stage for this reason, and suggest that these could be included in protocols.

The rest of the paper is set out as follows. In section 2, we explain how to derive the power of the studies that contribute to a meta‐analysis, so that the study specific powers can subsequently be computed and compared to those from a meta‐analysis that combines these studies. In section 3, we examine the implications for the fixed‐effect model, where we quickly find that a fixed‐effect meta‐analysis necessarily results in an increase in power. In section 4, we examine the random‐effects model and propose a new way to derive the average study specific power and 3 ways to derive the power of random‐effects meta‐analyses. In section 5, we assess the performance of the methods proposed in section 4. In section 6, we explore the study specific and meta‐analysis powers empirically in a large database from Cochrane. Together the findings in sections 5 and 6 enable us to reach some important conclusions that we discuss in section 7.

## 2 THE POWER OF THE INDIVIDUAL STUDIES THAT CONTRIBUTES TO THE META‐ANALYSIS

In this section, we derive the power of the individual studies that contribute to the meta‐analysis. At this stage, we make no use of meta‐analysis methodology, because we make no assumptions about how the true treatment effects for each study relate to each other.

We let *μ*_*i*_ denote the true effect in study *i* and let *k* denotes the number of studies. We let *Y*_*i*_ denotes this study's estimate of *μ*_*i*_ and let *σ*_*i*_ denotes the corresponding standard error. These standard errors are usually estimated in practice but treated as if fixed and known in analysis. We suppress the fact that the within‐study standard errors are estimated prior to performing the meta‐analysis, and so write *σ*_*i*_ instead of ${\hat{\sigma }}_{i}$. We also use normal within‐study approximations $Y_{i}∼N(\mu_{i},\sigma_{i}^{2})$, as is conventional in meta‐analysis and common when analysing data from individual trials. We assume that 2‐tailed hypothesis tests are used throughout. We make no attempt to distinguish “accepting the null hypothesis” and “not rejecting the null hypothesis” and other more subtle issues related to the interpretation of hypothesis and significance testing.

From the standard textbook theory of hypothesis testing using a normally distributed estimate with known standard error (eg, Matthews and Farewell,^17^ their chapter 8), the test statistic *H*_0_:*μ*_*i*_=*μ*_0_ versus *H*_1_:*μ*_*i*_≠*μ*_0_ in the *i*th study is given by *Z*_*i*_=(*Y*_*i*_−*μ*_0_)/*σ*_*i*_; typically we set *μ*_0_=0 to test for no effect. Under the null hypothesis, *H*_0_:*μ*_*i*_=*μ*_0_, *Z*_*i*_∼*N*(0,1). Under the alternative hypothesis *Z*_*i*_∼*N*(*δ*_*i*_/*σ*_*i*_,1), where *δ*_*i*_=*μ*_*i*_−*μ*_0_. The null hypothesis is rejected using a 2‐tailed test by the *i*th study if $|Z_{i}|⩾Z_{a}$, and this hypothesis is accepted if |*Z*_*i*_|<*Z*_*a*_, where *Z*_*a*_ is a suitable critical value from a standard normal distribution; *Z*_*a*_=1.96 gives the conventional 5% significance level that we will assume is used in our investigations below. The probability of accepting the null hypothesis is therefore equal to Φ(*Z*_*a*_−*δ*_*i*_/*σ*_*i*_)−Φ(−*Z*_*a*_−*δ*_*i*_/*σ*_*i*_), where Φ(·) is the standard normal cumulative distribution function. Hence, the power is given by the probability of correctly rejecting the null hypothesis when it is false, which is

(1) $$\begin{array}{ccc} \beta_{i}(\delta_{i},\sigma_{i}) & =1+\Phi (-Z_{a}-\delta_{i}/\sigma_{i})-\Phi \left(Z_{a}-\delta_{i}/\sigma_{i}\right) & \\ & =1+\Phi (-Z_{a}+\delta_{i}/\sigma_{i})-\Phi (Z_{a}+\delta_{i}/\sigma_{i}). \end{array}$$

The power varies from one study to the next, depending on the study specific *δ*_*i*_ and *σ*_*i*_. Large powers are obtained for studies, where *δ*_*i*_ is of large magnitude, and *σ*_*i*_ is small. This reflects the intuition that we will be likely to detect effects when they are large and/or when studies provide a large amount of information.

### 2.1 The probability of rejecting the null hypothesis and inferring the correct directional effect

It may also be of interest to evaluate the probability of rejecting the null hypothesis and inferring that *μ*_*i*_>*μ*_0_ (that is, observing *Y*_*i*_>*μ*_0_), so that the probability of detecting an effect in the correct direction can be calculated. This type of calculation is also necessary for computing the power of 1‐tailed tests. This probability is given by

$$\beta_{i}^{+}(\delta_{i},\sigma_{i})=\Phi (-Z_{a}+\delta_{i}/\sigma_{i}).$$

All the other methods for calculating powers below are also easily modified to calculate the probability of rejecting the null hypothesis and also inferring a particular directional effect. Hence, we do not give further explicit details of how to modify our methods in this way. Those who would prefer not to include the “type III error” (correctly rejecting the null hypothesis but inferring the wrong directional effect) in the power are particularly likely to modify the methods in this manner. We welcome the use of this and other modifications that analysts might wish to adopt; for example, it has been proposed to replace *Z*_*a*_ with an appropriate quantile from a t distribution when performing meta‐analyses, and this is another easy and obvious way to modify some of the methods that follow. We however allow the “type III error” to contribute to the power because this is so ubiquitous in the established literature that we discuss in the introduction that we do not attempt to challenge this convention here. However, we are sympathetic to the position that the “type III error” should not be included in the power.

## 3 THE FIXED‐EFFECT MODEL

The power for each individual study is easily calculated as explained in section 2. We now examine the power of meta‐analyses that combine such studies, so that the study specific and the meta‐analysis powers can be compared. The fixed‐effect (or common‐effect) model assumes that there is no between‐study variation, so that *μ*_*i*_=*μ*, and *δ*_*i*_=*δ*=*μ*−*μ*_0_ for all *i*. This means that we assume that $Y_{i}∼N(\mu ,\sigma_{i}^{2})$, and calculating powers of hypothesis tests are straightforward.

### 3.1 The power of the individual studies

Upon substituting *δ*_*i*_=*δ*, in Equation (1), the study‐specific powers under the fixed‐effect model are

(2) $$\begin{array}{ccc} \beta_{i}(\delta ,\sigma_{i}) & =1+\Phi (-Z_{a}-\delta /\sigma_{i})-\Phi \left(Z_{a}-\delta /\sigma_{i}\right) & \\ & =1+\Phi (-Z_{a}+\delta /\sigma_{i})-\Phi (Z_{a}+\delta /\sigma_{i}). \end{array}$$

The powers of the studies in Equation (2) are not identical because the *σ*_*i*_ differ. To obtain an average study specific power, we approximate the distribution of the *σ*_*i*_ with their empirical distribution. Hence, the average study specific power is

(3) $$\bar{\beta }(\delta ,\sigma )=\frac{1}{k}\overset{k}{\underset{i=1}{\sum }}\left\{1+\Phi (-Z_{a}+\delta /\sigma_{i})-\Phi (Z_{a}+\delta /\sigma_{i})\right\},$$

where ***σ*** is a vector containing the *σ*_*i*_. We present the average study specific power as a useful descriptive statistic that describes an important feature of the evidence base. Other summaries of the empirical distribution of the study specific powers, or indeed the meta‐analysis powers below, could also be presented as descriptive statistics.

### 3.2 The power of fixed‐effect meta‐analyses

Under the assumption of a fixed‐effect model, there is a single parameter *μ* to estimate, and the pooled estimate is given by $\hat{\mu }=\sum \sigma_{i}^{-2}Y_{i}/\sum \sigma_{i}^{-2}$, where $\hat{\mu }∼N\left(\mu ,V_{F}=1/\sum \sigma_{i}^{-2}\right)$. Hence, the power of the 2‐sided hypothesis test *H*_0_:*μ*=*μ*_0_ is given by expression (2) with *σ*_*i*_ replaced by $\sqrt{V_{F}}$, which is

(4) $$\begin{array}{ccc} \beta_{F}(\delta ,\sigma ) & =1+\Phi (-Z_{a}-\delta /\sqrt{V_{F}})-\Phi \left(Z_{a}-\delta /\sqrt{V_{F}}\right) & \\ & =1+\Phi (-Z_{a}+\delta /\sqrt{V_{F}})-\Phi (Z_{a}+\delta /\sqrt{V_{F}}). \end{array}$$

This is equivalent to Equation (8) of Hedges and Pigott.^15^

Assuming that there is more than a single study in the meta‐analysis, it is straightforward to show that $\sqrt{V_{F}}<\sigma_{i}$ for all *i*. Using this fact, it is then straightforward to show that the power of the fixed effect meta‐analysis is greater than all the study specific powers in Equation (2). A similar analysis shows that the fixed‐effect meta‐analysis also possesses more power than the individual studies in the context of 1‐sided hypothesis tests, provided that *μ* lies in the direction of the alternative hypothesis. Of course if there is a single study then the study specific and meta‐analysis powers are the same.

This analysis shows that in the case of a fixed‐effects model, the claim that “meta‐analyses increase power” is completely justifiable. This conclusion was also reached by Cohn and Becker,^13^but we also give details here to motivate our analysis of the random‐effects model, which provides our main interest.

## 4 THE RANDOM‐EFFECTS MODEL

The analyses of individual studies (section 2) and the fixed‐effect model (section 3) are straightforward. However, matters are more complicated under the random‐effects model. The random‐effects model relaxes the assumption that *μ*_*i*_=*μ* for all *i* and instead assumes *μ*_*i*_∼*N*(*μ*,*τ* ^2^), so that *δ*_*i*_∼*N*(*δ*,*τ* ^2^). If *τ* ^2^=0, then we have *δ*_*i*_=*δ*, and we recover the fixed‐effect model as a special case. The random‐effects model is often presented as a slight modification of the fixed‐effect model. This is because *τ* ^2^ is typically estimated and then treated as fixed and known in analysis, so that $\sigma_{i}^{2}$ is replaced by $\sigma_{i}^{2}+{\hat{\tau }}^{2}$ in analysis. However, this does not take into account the fact that *τ* ^2^ is estimated, and the uncertainty in *τ* ^2^ is considerable in typical meta‐analyses with few studies.^18^, ^19^, ^20^ Another complication when comparing powers is that the powers of the individual studies that contribute to a random‐effects meta‐analysis now depend on random *δ*_*i*_.

### 4.1 The average power of the individual studies

Now that *δ*_*i*_ is a random variable, we can obtain the average study specific power $\beta (\delta_{i},\sigma_{i}^{2})$ by taking the expectation of *β*(*δ*_*i*_,*σ*_*i*_) (from Equation  (2)) over the joint distribution of (*δ*_*i*_,*σ*_*i*_). For the fixed‐effect model, only the *σ*_*i*_ differed across studies, and we took this expectation over their empirical distribution. Under the random‐effects model, we have *δ*_*i*_∼*N*(*δ*,*τ* ^2^), and we continue to approximate the distribution of *σ*_*i*_ with their empirical distribution, where we further assume that *δ*_*i*_ and *σ*_*i*_ are independent. In situations where an association between study specific estimates and their precision is observed then this is generally attributed to small study effects or publication bias; we assume that no such phenomena are present.

In the web supplementary materials, we show that the average power of an individual study that contributes to the random‐effects meta‐analysis is

(5) $$\begin{array}{ccc} \bar{\beta }(\delta ,\tau^{2},\sigma ) & =\frac{1}{k}\sum_{i=1}^{k}\left\{1+\Phi \left((-Z_{a}\sigma_{i}+\delta )/\sqrt{\sigma_{i}^{2}+\tau^{2}}\right)\right. & \\ & \;-\left.\Phi \left((Z_{a}\sigma_{i}+\delta )/\sqrt{\sigma_{i}^{2}+\tau^{2}}\right)\right\}. \end{array}$$

If *τ* ^2^=0, then Equation (5) reduces to Equation (3). Equation (5) shows how the between‐study variance affects the average study power.

### 4.2 Fitting the random‐effects model

The application of the random‐effects model requires an estimate of the between‐study variance, and many estimators are available.^20^ The simplest and most commonly used estimate of *τ* ^2^ in Equation (6) is the DerSimonian and Laird^10^ estimate. We will assume that this estimator is used throughout, to examine power in the current statistical climate, but we come back to this issue in section 4.3.3 and the discussion. This uses the *Q* statistic,

$$Q=\overset{k}{\underset{i=1}{\sum }}w_{i}{\left(\;y_{i}-\bar{y}\right)}^{2},$$

where $w_{i}=\sigma_{i}^{-2}$, $\bar{y}=\overset{k}{\underset{i=1}{\sum }}w_{i}y_{i}/\overset{k}{\underset{i=1}{\sum }}w_{i}$. Under the assumptions of the random‐effects model we have

$$E[Q]=(k-1)+\left(S_{1}-\frac{S_{2}}{S_{1}}\right)\tau^{2},$$

where $S_{r}=\overset{k}{\underset{i=1}{\sum }}w_{i}^{r}$, which provides the DerSimonian and Laird estimate

$${\hat{\tau }}^{2}=\max\left(0,\frac{Q-(k-1)}{S_{1}-S_{2}/S_{1}}\right).$$

The estimate of the overall treatment effect is then given by $\hat{\mu }=\overset{k}{\underset{i=1}{\sum }}w_{i}^{*}y_{i}/\overset{k}{\underset{i=1}{\sum }}w_{i}^{*}$, where $w_{i}^{*}={\left({\hat{\sigma }}_{i}^{2}+{\hat{\tau }}^{2}\right)}^{-1}$, and the distribution of $\hat{\mu }$ is approximately $\hat{\mu }∼N\left(\mu ,{\left(\sum_{i=1}^{k}w_{i}^{*}\right)}^{-1}\right)$. The resulting test statistic for testing *H*_0_:*μ*=*μ*_0_ is given by *T*, the ratio of $\hat{\mu }-\mu_{0}$ and its approximate standard error, which can be written as

(6) $$T=\frac{\overset{k}{\underset{i=1}{\sum }}\frac{Y_{i}-\mu_{0}}{\sigma_{i}^{2}+{\hat{\tau }}^{2}}}{\sqrt{\sum_{i=1}^{k}\frac{1}{\sigma_{i}^{2}+{\hat{\tau }}^{2}}}}.$$

The evaluated test statistic *T* is then conventionally compared to an appropriate percentile of a standard normal distribution.

### 4.3 The power of random‐effects meta‐analyses

The distribution of *T* in Equation (6) is, at best, very difficult to obtain analytically so that suitable power formulae are harder to derive than for the fixed‐effect model. We therefore suggest 3 approaches for evaluating the power of the test based on Equation (6), that have different advantages and disadvantages. The first 2 methods require values of *δ* and *τ* ^2^, and the within‐study variances, and are very closely related. The first method is a well established approximate analytical approach, and the second is a more computationally expensive numerical analogue of the first method that allows for the uncertainty in estimates of *τ* ^2^. The third method assumes that all studies are the same size and requires just the number of studies, the proportion of variation that is due to between‐study heterogeneity (which we will denote as *I* ^2^, where this is the quantity that the *I* ^2^ statistic proposed by Higgins and Thompson^21^ estimates) and a noncentrality parameter that depends on *δ*, the number of studies and the study size.

#### 4.3.1 An analytical approach

When applying the random‐effects model, we typically apply the fixed‐effect methodology where $\sigma_{i}^{2}$ is replaced by $\sigma_{i}^{2}+\tau^{2}$, where *τ* ^2^ is usually taken to be its estimated value. Hence, the power can be taken to be as described in Equation (4) for the fixed‐effect model but where *V*_*F*_ is replaced with $V_{R}=1/\sum {\left(\sigma_{i}^{2}+\tau^{2}\right)}^{-1}$, which gives

(7) $$\begin{array}{ccc} \beta_{R}(\delta ,\tau^{2},\sigma ) & =1+\Phi (-Z_{a}-\delta /\sqrt{V_{R}})-\Phi \left(Z_{a}-\delta /\sqrt{V_{R}}\right) & \\ & =1+\Phi (-Z_{a}+\delta /\sqrt{V_{R}})-\Phi (Z_{a}+\delta /\sqrt{V_{R}}), \end{array}$$

where we now use the subscript “R” to emphasise that this is the power under the random‐effects model. This is equivalent to eq. 24 of Hedges and Pigott,^15^ who suggest using this equation with the estimated between‐study variance and also what they refer to as small, medium, and large between‐study heterogeneities. We will therefore regard this type of approach as the conventional method for power analysis under the random‐effects model. The main advantages of this standard approach are its computational and conceptual simplicity. The main disadvantage of this approach is that it does not take into account statistical properties of, and so the uncertainty in, the estimation of *τ* ^2^ and the implications this has for making inferences about the average effect.

#### 4.3.2 A Monte Carlo approach

To allow for the uncertainty in *τ* ^2^ when making inferences about the average effect, but otherwise use the same type of approach as in Equation (7), an analogous Monte Carlo method can be used. To conveniently use Monte Carlo methods for evaluating the power of a random‐effects meta‐analysis, we define $X_{i}=Y_{i}-\mu_{0}∼N(\delta ,\sigma_{i}^{2}+\tau^{2})$, so that Equation (6) becomes

(8) $$T=\frac{\overset{k}{\underset{i=1}{\sum }}\frac{X_{i}}{\sigma_{i}^{2}+{\hat{\tau }}^{2}}}{\sqrt{\sum_{i=1}^{k}\frac{1}{\sigma_{i}^{2}+{\hat{\tau }}^{2}}}},$$

where, because the estimation of *τ* ^2^ is location invariant, values of *X*_*i*_ can be used instead of *Y*_*i*_ when computing *Q* and calculating ${\hat{\tau }}^{2}$ in Equation (8). Hence, we can obtain the power of the random‐effects meta‐analysis, for true values of *δ* and *τ* ^2^ and a set of within‐study variances, by simulating many meta‐analyses as $X_{i}∼N(\delta ,\sigma_{i}^{2}+\tau^{2})$, *i*=1,⋯*k*, and then using standard meta‐analysis software to perform random‐effects meta‐analyses using these outcome data. The *metafor* package and the command *rma.uni* will be used with the “DL”^10^ option for this purpose throughout. Then the proportion of simulated random‐effects meta‐analyses that are statistically significant at the appropriate level gives the power denoted as *β*_*R*_(*δ*,*τ* ^2^,***σ***), but obtained differently, in Equation (7).

The difference between this approach and the previous one is that it clearly distinguishes between the true and estimated *τ* ^2^. Although this Monte Carlo method requires the analyst to determine the true value of *τ* ^2^ to use in the power calculation, the estimated between‐study variance is used when computing the simulated test statistics that are used to determine the power of the test. In meta‐analyses with large numbers of studies, the approximation $\tau^{2}={\hat{\tau }}^{2}$ is appropriate when making inferences about the average effect, and we will see below that allowing for the uncertainty in the estimated between‐study variance becomes unimportant in meta‐analyses with very large numbers of studies. The advantage of this approach is that it allows for the uncertainty in the estimated between‐study variance when making inferences about the average effect, and so can be expected to provide more accurate powers. The disadvantage of this method is that it requires simulation and so is computationally more expensive and subject to Monte Carlo error.

#### 4.3.3 An analytical approach assuming that all studies are the same “size”

It is convenient to have a formula for the power as in the first method above, and yet also take into account the uncertainty in *τ* ^2^ when making inferences about the average effect, as in the second method above. To facilitate such an analytical result, we consider the artificial special case where all studies are the same “size” ( $\sigma_{i}^{2}=\sigma^{2}$ for all *i*; this means that all studies provide the same amount of information). We show in the web supplementary materials that the cumulative distribution function of *T* is given by

$$P(T⩽t)\;=\;\Gamma_{1}\;\left(\frac{k-1}{2},\frac{\left(1-I^{2})(k-1\right)}{2}\right)\Phi \left((t-\Delta )\sqrt{1-I^{2}}\right)$$

(9) $$+2(k-1)\int_{\sqrt{1-I^{2}}}^{\infty }x\Phi \left(tx-\Delta \sqrt{1-I^{2}}\right)\chi_{k-1}^{2}\left((k-1)x^{2}\right)dx,$$

where *I* ^2^=*τ* ^2^/(*σ* ^2^+*τ* ^2^), $\Delta =\delta \sqrt{k}/\sigma$ is a noncentrality parameter, $\chi_{k-1}^{2}(\cdot )$ is the probability density function of the *χ* ^2^ distribution with (*k*−1) degrees of freedom, and we define the incomplete Gamma function:

$$\Gamma_{1}(a,x)=\frac{1}{a}\int_{0}^{x}t^{a-1}\exp(-t)dt.$$

Although we assume the use of the DerSimonian and Laird estimator of *τ* ^2^ in our empirical investigation below, as we also explain in the web supplementary materials, the DerSimonian and Laird, REML and Paule Mandel estimators coincide when all variances are the same. See Veroniki et al.^20^ for full details of these and many other estimators. Hence, the results in this section apply whenever any of these 3 very popular estimators of *τ* ^2^ are used.

Then by taking, for example, $\sigma =\hat{\sigma }$, where ${\hat{\sigma }}^{2}$ is the typical within‐study variance used by Higgins and Thompson,^21^

(10) $${\hat{\sigma }}^{2}=\frac{(k-1)\sum_{i=1}^{k}\sigma_{i}^{-2}}{{\left(\sum_{i=1}^{k}\sigma_{i}^{-2}\right)}^{2}-\sum_{i=1}^{k}\sigma_{i}^{-4}},$$

we can consider an analogous meta‐analysis to a real one where all within‐study variances are equal to this representative value. Since all the other parameters are unconstrained, this analogous meta‐analysis has the same essential features (overall effect, between‐study variance, number of studies) that drive the power in the real meta‐analysis. Γ_1_(*a*,*x*) is provided by statistics packages, and we can evaluate the integral in Equation (9) numerically to obtain the cumulative distribution function of the test statistic. The probability of accepting the null hypothesis is then obtained as $P(T⩽Z_{a})-P(T⩽-Z_{a})$, and the power of the random‐effects meta‐analysis is then obtained by subtracting this probability from 1. This method gives the same power as the second method when all studies are the same size (although the second method is subject to Monte Carlo error), and so also computes the power denoted as *β*_*R*_(*δ*,*τ* ^2^,***σ***), but obtained differently, in Equation (7).

The main advantage of this method is that it takes into account the uncertainty in *τ* ^2^ without resorting to simulation. As we will explain below, the quantities required for this method are more amenable to researchers performing power calculations at the planning stage. The main disadvantage of this approach is that it requires considering an artificial special case, but this type of special case was also used by Hedges and Pigott (2001)^15^ to make the first method more accessible to applied analysts.

## 5 ASSESSING THE PERFORMANCE OF THE 3 METHODS FOR CALCULATING META‐ANALYSIS POWER UNDER THE RANDOM‐EFFECTS MODEL

We now have 3 main proposals for computing the power of random‐effects meta‐analyses. The main differences between the methods are whether or not uncertainty in *τ* ^2^ is taken into account, and whether or not all studies are assumed to be the same size. Hence, the relative importance of these factors are crucial in determining which method should be recommended in practice.

### 5.1 The implications of allowing for the uncertainty in the estimated between‐study variance

The more conventional method for power analysis in random‐effects meta‐analysis, described in section 4.3.1, does not allow for the uncertainty in the estimation of *τ* ^2^ when making inferences about the average effect. To investigate how important this consideration is for meta‐analysis power calculations, we will initially consider the case where all studies are the same size so that we can perform the investigation analytically.

When all studies are the same size, so that $\sigma_{i}^{2}=\sigma^{2}$, in Equation (7), we have $V_{R}=(\sigma^{2}+\tau^{2})/k$, so that Equation (7) becomes

(11) $$\beta_{R}(\Delta ,I^{2})=1+\Phi (-Z_{a}+\Delta \sqrt{1-I^{2}})-\Phi (Z_{a}+\Delta \sqrt{1-I^{2}}).$$

This means that power calculations that do not allow for the uncertainty in *τ* ^2^, from Equation (11), can be compared to those that do (section 4.3.3) for this special case.

### 5.2 An important comparison

We have emphasised in the introduction that the meta‐analysis and study‐specific hypothesis tests involve different types of hypotheses: meta‐analyses test for evidence of a population average effect but study‐specific tests instead test for the evidence of an effect in the individual study. This difference can be made explicit by examining the case where *k*=1 and *τ* ^2^ is treated as fixed and known. Under the fixed‐effect model, the study specific and meta‐analysis powers are the same because then the single within‐study variance $\sigma_{1}^{2}$ is equal to *V*_*F*_. However, under the random‐effects model, writing $\sigma_{1}^{2}=\sigma^{2}$, the “average” study‐specific power (Equation (5)) can be written as

(12) $$\;1+\Phi \;\left(\;-Z_{a}\sqrt{\;1\;-I^{2}}\;+\;\frac{\delta }{\sqrt{\sigma^{2}+\tau^{2}}}\;\right)-\Phi \;\left({\;Z}_{a}\;\sqrt{\;1\;-I^{2}}\;+\;\frac{\delta }{\sqrt{\sigma^{2}+\tau^{2}}}\;\right),$$

and the meta‐analysis power (Equation (11)) can be written as

(13) $$1+\Phi \left(-Z_{a}+\frac{\delta }{\sqrt{\sigma^{2}+\tau^{2}}}\right)-\Phi \left(Z_{a}+\frac{\delta }{\sqrt{\sigma^{2}+\tau^{2}}}\right).$$

Comparing Equations (12) and (13), we see that these powers are of the same standard form resulting from a 2‐tailed hypothesis test involving a single normally distributed random variable. However, the study‐specific hypothesis test in Equation (12) uses critical values of $\pm Z_{a}\sqrt{1-I^{2}}$, whereas the meta‐analysis hypothesis test uses the more usual standard normal critical values ±*Z*_*a*_. We have $I^{2}⩾0$ so that $\sqrt{1-I^{2}}⩽1$, which means that the power of the study‐specific hypothesis test is equivalent to the meta‐analysis hypothesis test but where the study‐specific hypothesis test has used smaller critical values. This means that the study‐specific power in Equation (12) is greater than the random‐effects meta‐analysis power in Equation (13) if between‐study heterogeneity is present. This analysis clarifies the different natures of the 2 types of hypothesis tests and also indicates that sufficient numbers of studies will be needed for the random‐effects meta‐analysis to achieve greater power than the individual studies that contribute to it.

### 5.3 Comparing the powers from calculations that do, and do not, take into account the uncertainty in τ ^2^

The powers calculated from Equation (11) depend only on the noncentrality parameter $\Delta =\delta \sqrt{k}/\sigma$ and *I* ^2^=*τ* ^2^/(*σ* ^2^+*τ* ^2^). However the powers calculated from Equation (9), and as explained in section 4.3.3., further depend on *k*, where this dependence can also be conveniently expressed in the degrees of freedom (*k*−1). It is obvious that Equation (11) does not depend on the sign of Δ, and only on its magnitude |Δ|, because Φ(*z*)=1−Φ(−*z*). It is perhaps less obvious that this is also the case in powers calculated in the way explained in section 4.3.3, but the same identity also establishes this. Hence, we only need consider, for example, positive Δ, because the results for −Δ are the same. This is in any case intuitively obvious because we perform conventional 2‐tailed tests, so that the power depends only on the magnitude of *δ* and so on the magnitude of Δ.

**Figure 1:**
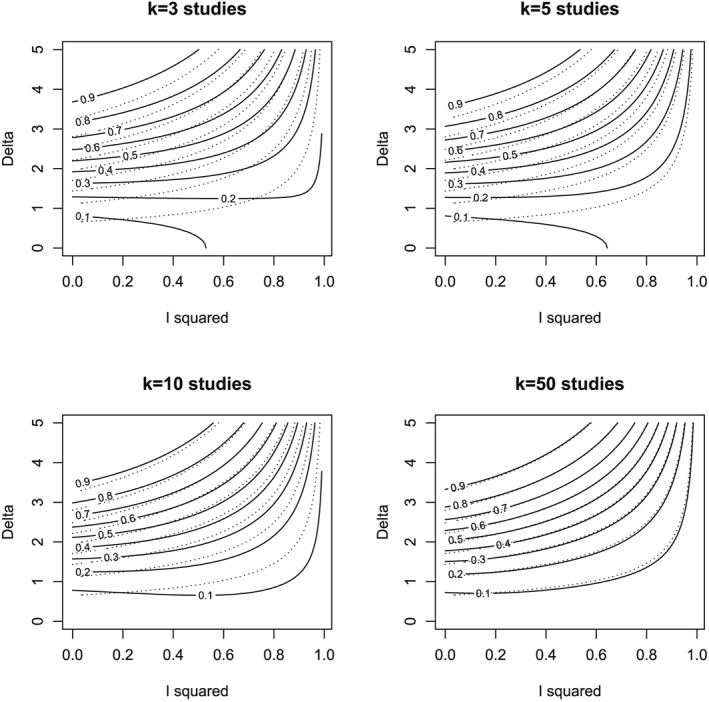
The implications of ignoring the uncertainty in ${\hat{\tau }}^{2}$ when performing power calculations. This figure explores the special case where all studies are the same size. The 4 plots show the power of the standard random‐effects model's hypothesis test for k=3, 5, 10, and 50 studies, as a function of Δ and I ^2^. These plots allow for the fact that the between‐study variance is estimated in practice. The dotted lines on each plot show the power of this test when ignoring the uncertainty in the estimated between‐study variance, or equivalently as the sample size tends towards infinity. Note that Δ is an increasing function in k, so that as the sample size increases Δ corresponds to a decreasing effect δ

In Figure 1, we show the contour plots of the resulting powers in Δ and *I* ^2^, obtained using Equation (9). In each plot, we show power contours at 0.1, 0.2, ⋯, 0.9, and we also show the corresponding contours using Equation (11) as dotted lines (without labelling them, to avoid cluttered figures). As the sample size *k* increases, the results using Equation (9) to calculate the power become more similar to those from Equation (11), and for inordinately large *k*(1000 say, results not shown), the powers obtained using Equation (9) and from Equation (11) become indistinguishable. This is because as the sample size increases, the uncertainty in *τ* ^2^ becomes negligible. For small *k* and large *I* ^2^, much smaller Δ are apparently required to achieve low power from Equation (9) than Equation (11), but this is an artifact of the standard methods for random‐effects meta‐analysis being anticonservative in small samples where the heterogeneity is severe; the random‐effects model's hypothesis test possesses considerably less than its nominal significance level in such situations, which results in artificially small Δ to achieve powers that are only slightly greater than the nominal significance level. Note also that Δ is an increasing function in *k*. Hence, for a fixed effect *δ* and within‐study standard error *σ* ^2^, as *k* increases so does Δ. Hence, Δ corresponds to a smaller effect *δ* as *k* increases, and from Figure 1, we can see that the power to detect any fixed value of *δ* increases as the sample size *k* becomes larger.

Figure 1 shows that conventional methods for power analysis under the random‐effects model, that do not allow for the fact that *τ* ^2^ must be estimated, serve as a reasonable guide to the actual power, especially for large *k*. Hence, ignoring the uncertainty in *τ* ^2^ in the power calculation is not a very serious source of concern. However, small *k* is extremely common place in practice,^22^ and we can also see that the conventional method performs least accurately in such instances.

We also investigated this issue empirically using a database of 1991 meta‐analyses from the Cochrane Database of Systematic Reviews (Issue 1, 2008). We used the log risk ratio as the measure of treatment effect; we had access to the raw count data so that any differences in the method of analysis used in the individual reviews presented no difficulties. Most Cochrane reviews contain multiple meta‐analyses, corresponding to different pairwise comparisons of interventions and different outcomes examined. Davey et al^22^ classified each meta‐analysis by outcome type, the type of interventions compared and the medical specialty. Here, we use data on the first reported binary outcome meta‐analysis within each of the 1991 Cochrane reviews reporting at least 1 binary outcome meta‐analysis in the full database extracted by Davey et al.^22^ We performed retrospective power calculations for all 1991 random‐effects meta‐analyses using the analytical (section 4.3.1) and Monte Carlo (section 4.3.2) approaches, that do not, and do, allow for the uncertainty in *τ* ^2^, respectively. Now that we apply the random‐effects model to the Cochrane data, it is pertinent to recall that this model can be a quite crude approximation in practice. For example, see Hoaglin^23^ and Shuster and Walker^24^ for good discussions of this issue.

**Figure 2:**
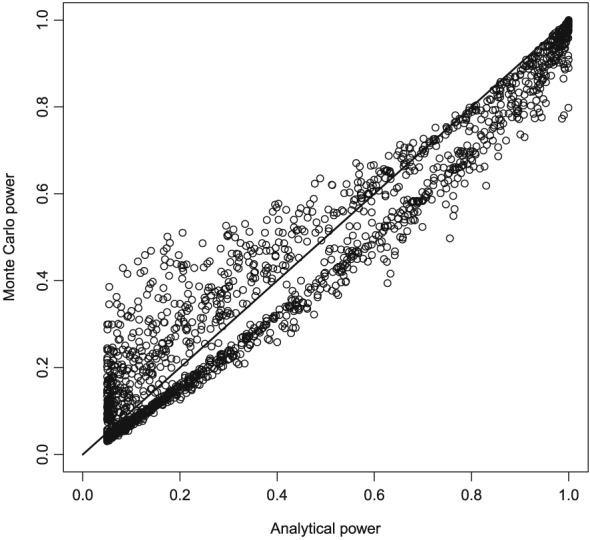
The implications of ignoring the uncertainty in ${\hat{\tau }}^{2}$ when performing power calculations. This figure shows the results of the empirical investigation of power in 1991 meta‐analyses. A line of equality is also shown

For each meta‐analysis, we took *δ* to be the absolute value of the random‐effects pooled estimate of the average relative risk (though its sign is irrelevant, as explained above) and *τ* ^2^ to be the DerSimonian and Laird estimate of the between‐study variance. By taking *δ* to be the (absolute) pooled estimate in this way, we perform retrospective power calculations for the null hypothesis that there is no treatment effect. We used 10 000 iterations when using the Monte Carlo approach, and the resulting 1991 pairs of powers are shown in Figure 2, where the powers obtained using the Monte Carlo method (section 4.3.2.) are shown on the vertical axis, and the powers obtained using the analytical approach (section 4.3.1.) are shown on the horizontal axis.

Figure 2 shows that the powers are in reasonable agreement. Hence ignoring the uncertainty in *τ* ^2^ in the analytical approach continues to appear not to be a very serious concern. Despite this, it is also evident that the power calculated from the Monte Carlo method can differ substantially from the analytical power. This is because the actual powers of meta‐analysis hypothesis tests differ from the powers obtained using methods that ignore the uncertainty in estimates of *τ* ^2^. The analytical powers in Figure 2 are bounded below by 0.05, but this is not the case for the Monte Carlo method because of the approximate nature of the standard random‐effects methods for meta‐analysis; when the between‐study variance is zero or very small, the standard methods are conservative so that actual powers of less than 0.05 are possible. More commonly, in other instances where the analytical power is very low, much higher powers using the Monte Carlo method are possible. This too is an artifact of the approximate nature of standard methods for random‐effects meta‐analysis, where if there are very small numbers of studies and very considerable between‐study variation, standard methods for random‐effects meta‐analysis are highly anticonservative and artificially high powers can be obtained; this is also evident in the top left hand plot in Figure 1 for *k*=3.

We conclude that it is desirable, but not essential, to use methods for power analysis under the random‐effects model that take into account the uncertainty in the estimation of *τ* ^2^ when making inferences about the average effect.

### 5.4 Using the method assuming all studies are the same “size” to serve as a guide for retrospective power calculations

The suggestion in section 4.3.3 was to use an analytical approach where all studies are the same size, where the within‐study standard error was taken to be the square root of corresponding typical within‐study variance suggested by Higgins and Thompson.^21^ This type of approach has been suggested previously (eg, Jackson and Bowden,^25^) for obtaining a guide to how methods for meta‐analysis perform. It is tempting to consider this type of approach in large scale empirical investigations, like the one below, because the analytical results are obtained almost instantly. But this computational ease comes at the price of using an analogous meta‐analysis to the one that has been observed to obtain an indication of the power in the real meta‐analysis. We therefore also investigated this particular issue carefully using the database of meta‐analyses from the Cochrane Database of Systematic Reviews.

**Figure 3:**
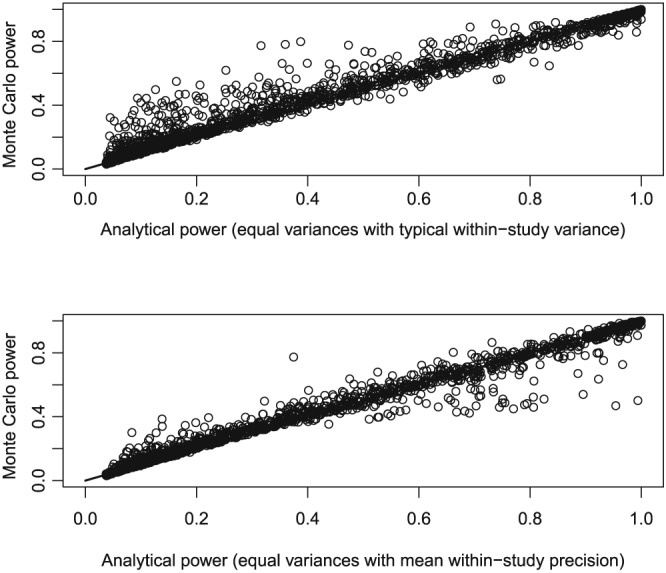
The implications of performing power calculations that assume all studies are the same size. This figure shows the results of the empirical investigation. The top figure shows the results taking all within‐study variances to be the typical within‐study variance in Equation (10), and the bottom figure takes $\sigma_{t}=k/\sum \sqrt{w_{i}}$. Lines of equality are also shown but are barely visible

Specifically, we compared the 1991 powers obtained using the Monte Carlo method (as explained in the previous section) to those from the analytical approach in section 4.3.3 (top plot in Figure 3). We can see that, in general, the analytical powers are in good agreement with those obtained using simulation and the observed distribution of the within‐study variances. Given the relative computational and conceptual simplicity of the analytical powers, Figure 3 provides strong evidence that it is more than adequate for giving a good indication of the power. However, the agreement between the 2 powers for some meta‐analyses is not so strong, and a good indicator of whether this is the case or not is of course the amount of variation in the within‐study variances; the analytical powers generally agree less well for meta‐analyses with the greatest variation in study sizes, as expected.

We also considered using alternatives to the typical within‐study variance (Equation (10)) as a measure of typical study size, such as the mean and median of the within‐study variances or the corresponding standard deviations. An alternative that also resulted in good agreement was taking the typical within‐study standard deviation to be the reciprocal of the mean of the within‐study “precisions,” ie, instead taking $\sigma_{t}=k/\sum \sqrt{w_{i}}$, and the corresponding results are shown in the bottom plot in Figure 3. However, no obvious alternative resulted in any visible improvement to the agreement level between the powers shown in Figure 3, which indicates that performing power calculations that take all studies to be the same size can give a reasonable indication of the power but also that powers of this type are not very accurate in every case.

In conclusion, it would appear to be desirable, but not essential, to take the distribution of the within‐study variances and the uncertainty in the estimation of *τ* ^2^ into account. The Monte Carlo method described in section 4.3.2 is computationally feasible and achieves both aims so it would seem reasonable to make the general recommendation that this method should be used for retrospective power calculations.

### 5.5 Using the methodology assuming all studies are the same “size” to perform power analyses at the planning stage

The method in Section 4.3.3. is, despite its limitations, useful for power calculations at the planning stage, where detailed knowledge of the distribution of the within‐study variances is unlikely to be available. We therefore suggest that this particular method should be considered when performing this type of power analysis. Here, the analyst specifies the anticipated number of studies, a typical within‐study variance and an *I* ^2^ statistic. The typical within‐study variance can be obtained from the relevant formulae for their calculation them and using information such as a representative study size. This is quite a lot of information to posit, and a variety of possibilities could be explored. Then figures like those in Figure 1 may be used to give an indication of the value of Δ, and hence *δ*, that is needed to obtain powers of interest. In the supplementary materials that accompany this paper, we provide R code to produce plots like those in Figure 1 with an arbitrary *k*, to facilitate power calculations at the planning stage in this way.

## 6 AN EMPIRICAL INVESTIGATION COMPARING META‐ANALYSIS AND STUDY SPECIFIC POWERS UNDER THE RANDOM‐EFFECTS MODEL

Now that we have determined the most suitable way to perform retrospective power calculations under the random‐effects model, we are able to compare the meta‐analysis and average study specific powers. Specifically, we will compare the powers obtained in Section 5 using the Monte Carlo method to the average study specific power from Equation (5) with the same values of *δ* and *τ* ^2^. By taking *δ* to be the pooled estimates, we therefore continue to assume that interest lies in testing the null hypothesis of no treatment effect.

The meta‐analysis power was found to be greater than the average study specific power in 303/609 (49.8%) random‐effects meta‐analyses where *k*=2. For *k*=3, this was 211/322 (65.5%); for *k*=4, this was 170/236 (72.0%); for *k*=5, this was 134/169 (79.3%); for *k*=6,7,8,9, this was 290/355 (81.7%); and for k⩾10, this was 263/300 (87.7%). We used these groups to provide reasonably large proportions of meta‐analyses in each group. A trend where the power increases with the number of studies is clear, as we should expect, although the observational nature of this conclusion should be emphasised because it does not control for other important factors such as the assumed average effect or the variance structures in the meta‐analyses that contribute to each of the 6 groupings. Under the strong assumption that our data are representative of meta‐analysis datasets, we estimate that random‐effects meta‐analysis results in an increase in power in 1371/1991 of meta‐analyses, which is just under 70%. However, it is clear that our sample of meta‐analyses contains many meta‐analyses where *k* is small; an increase in power when using random‐effects meta‐analysis is much more likely in subject areas where *k* is typically much larger.

Using the median, rather than the mean, study specific power in this comparison resulted in rather similar proportions of 303/609 (49.8%), 215/322 (66.8%), 173/236 (73.3%), 135/169 (79.9%), 302/355 (85.1%), and 269/300 (89.7%); using the median makes the power of the meta‐analysis look slightly better in this comparison. Instead using the maximum study specific power in this comparison resulted in proportions of 135/609 (22.2%), 107/322 (33.2%), 105/236 (44.5%), 88/169 (52.1%), 194/355 (54.6%), and 222/300 (74.0%). This suggests that in many meta‐analyses, the largest study will possess more power than the random‐effects meta‐analysis that it contributes to.

The overall picture from this empirical investigation is that if there are less than 5 studies then obtaining less power from the random‐effects meta‐analysis than from the individual studies that contribute to this meta‐analysis is quite likely in practice. Not only is statistical inference most difficult in this type of situation,^24^ but also it is less worthwhile in such cases. Most meta‐analysis powers are greater than the average study specific power however, so this investigation does not entirely discourage the use of meta‐analysis to obtain greater power. Despite this, our investigation certainly challenges the notion that meta‐analyses necessarily provide greater power.

## 7 DISCUSSION

We have investigated three different methods for performing power calculations for random‐effects meta‐analyses. We suggest that the Monte Carlo method should be used for retrospective power calculations because it is computationally feasible and allows for the uncertainty in the estimate of *τ* ^2^ and the distribution of the within‐study variances. We also suggest that our new approximate analytical method is very suitable power calculations at the planning stage. We suspect that advocates of random‐effects meta‐analysis will find the comparison of these two powers disappointing. Researchers working in very different application areas to those represented in the Cochrane database might argue that key parameters, such as the number of studies and effect sizes, differ considerably in their subject area, so that our empirical conclusion that meta‐analysis powers are disappointing does not necessarily apply in their work. Our analysis in section 3 demonstrates that meta‐analysis necessarily results in an increase in power when the data are correctly assumed to be homogenous. Combining this observation with our findings under the random‐effects model clarifies that between‐study heterogeneity has serious consequences for the power of meta‐analyses. The main difficulty for obtaining high power in random‐effects meta‐analysis would seem to be due to the presence of considerable between‐study heterogeneity. However, in small samples, there are further difficulties associated with estimating this parameter.

In meta‐analyses which lack power, precision in estimation is also lacking, and therefore the summary intervention effect is imprecisely estimated. Thorlund et al^26^ have warned that if meta‐analyses are performed too early, before enough studies are available, there is a danger that incorrect conclusions may be drawn. They recommend therefore that results from underpowered meta‐analyses are interpreted with caution. Trial sequential analysis methods proposed by Wetterslev et al^27^ can be used to evaluate whether or not a particular meta‐analysis contains enough information to be regarded as providing conclusive evidence. Ideally, extremely underpowered meta‐analyses should not be performed. However, we recognise that it is often difficult for researchers to know how many eligible studies provide data for any given outcome until most of the reviewing work has been performed, and at that stage withholding the results might result in reporting bias. In some settings, a lack of power is caused by high observed heterogeneity rather than by the number of studies, and this is even more difficult to predict in advance.

We have assumed that the conventional DerSimonian and Laird method for random‐effects meta‐analysis is used throughout. Calculating power using further alternative methods for random‐effects meta‐analysis is also possible, for example, using an altenative estimator of the between‐study variance.^20^ The Monte Carlo method that we advocate for retrospective power calculations can easily be adapted for other *τ* ^2^ estimators, and our approximate analytical method described in section 4.3.3 applies to a variety of these estimators, as we have explained. Other modifications and small sample corrections have been suggested (eg)^28^, ^29^ which inevitably have some implications for the power. For example, the artificially small values of Δ that achieve low powers, as seen in Figure 1, will not result in this difficulty when using the Hartung and Knapp modification in conjunction with common estimators of *τ* ^2^ when all studies are the same size. This is because the modification results in exact inference for this special case under the random‐effects model.^30^ Investigating the power when using more sophisticated methods is an important avenue for further research. In particular, for binary outcome data such that from the Cochrane database, methods that use binomial within‐study distributions^31^, ^32^ are in principle preferable to those that we have used here. However, the power formulae that we have provided have the merit of being simple and transparent and, provided that they can be shown to represent the amount of information and so the power when using more sophisticated methods, they may prove valuable long after current methodologies for meta‐analysis, such as the DerSimonian and Laird method, might be consigned to history.

Our methodology allows for the testing that the true effect is any value *μ*_0_, but in our empirical investigations, we have only explored the null value *μ*_0_=0. There is also interest in testing for clinically significant effects, and indeed we suggest that this should be considered more often in application. However, the testing of the null hypothesis that there is no treatment effect is so ubiquitous in application that we have restricted our investigation to this special case. Investigating meta‐analysis and study specific powers to detect other effects of interest could form the subject of future work.

There are many powerful and persuasive reasons for performing meta‐analyses; the desire to increase the power to detect an effect is just one such reason. Our investigations show that random‐effects meta‐analyses can achieve this aim and that they generally do. Provided that sufficient numbers of studies can be found then a gain in power is of course assured. However, we have found that the powers of real random‐effects meta‐analyses compare less favourably to the powers of the studies that contribute to them than many might suppose. As we also pointed out in the introduction, the standard meta‐analysis methods fail to provide the nominal significance level. We have not taken this into account, which means that some of the power that we have attributed to random‐effects meta‐analyses is likely to be artificial and due to using statistical methods that are often anticonservative. Hence, the observation that standard methods for meta‐analysis are merely approximate strengthens our case that random‐effects meta‐analyses possess less power, relative to the studies that contribute to them, than many might otherwise suppose. Our findings in no way diminish the valuable impact that systematic reviews and meta‐analyses have had, but do lead us to conclude that the notion that meta‐analyses necessarily increase power is one that we should be much more critical of.

In summary, we have provided new methods for power analysis for random‐effects meta‐analysis and have investigated how the power of this type of meta‐analysis compares to that of the studies that contribute outcome data. We hope that our new methods are a useful addition to the literature and that this article will serve to emphasise the importance of considerations of power in meta‐analysis. Finally, we hope that our empirical investigations will make applied analysts think more critically about whether random‐effects meta‐analyses, when applied to highly heterogeneous datasets with very few studies, are likely to provide more power than individual studies.

## Supporting information

Supplementary material

Appendix one
Appendix two
