# Supplementary material for: Power analysis for random‐effects meta‐analysis
Source: Res Synth Methods. 2017 Apr 4;8(3):290–302. doi: 10.1002/jrsm.1240 (PMC5590730; doi:10.1002/jrsm.1240)
Supplement: Supplementary file 1 — Supplementary material [file JRSM-8-290-s001.docx]

**Supplementary material**

The “make_contour” function produces a contour plot like those shown in Figure 1. Contour plots of this type are intended to be used for power calculations that are performed at the planning stage.

make_contour=function (n, main)

{

I2s=(0:99)/100

NCP=(0:50)/10

answers=matrix(nrow=length(I2s), ncol=length(NCP))

Hedges=answers

for(i in 1:length(I2s))

for(j in 1:length(NCP))

{

results=the_power(n, NCP[j],I2s[i])

answers[i,j]=results$power_DL

Hedges[i,j]=results$power_H

}

contour(I2s, NCP, answers, levels=(1:9)/10, xlab="I squared", ylab="Delta", main=main)

contour(I2s, NCP, Hedges, lty=3, add=TRUE,levels=(1:9)/10, labels=rep(" ", 9))

}

the_power= function (n, NCP, I2)

{

Z=qnorm(0.975)

power_DL=1-(CDF(Z, n, NCP, I2) - CDF(-Z, n, NCP, I2))

# power_DL is the power using the analytical approach assuming all studies are the same size

power_H=1 + pnorm(-Z+NCP*(1-I2)^0.5) - pnorm(Z+NCP*(1-I2)^0.5)

# power_H is Hedges and Pigott when all studies are the same size

return(list(power_DL=power_DL, power_H=power_H))

}

CDF= function (t, n, NCP, I2)

{

dof=n-1

constant=(1-I2)^0.5

part1=pgamma(dof*(1-I2)/2, dof/2)*pnorm((t - NCP)*constant)

part2=2*dof*integrate(func, Z=t, dof=dof, NCP=NCP, I2=I2, lower=constant, upper=Inf, rel.tol = 0.00000000000001)$value

part1+part2

}

func= function (x, Z, dof, NCP, I2)

{

x*pnorm(Z*x-NCP*(1-I2)^0.5) * dchisq(dof* x^2, dof)

}

make_contour(3, "k=3 studies")

# Top left hand plot in Figure 1.
