# Supplementary material for: Power analysis for random‐effects meta‐analysis
Source: Res Synth Methods. 2017 Apr 4;8(3):290–302. doi: 10.1002/jrsm.1240 (PMC5590730; doi:10.1002/jrsm.1240)
Supplement: Supplementary file 2 — Appendix one Appendix two [file JRSM-8-290-s002.pdf]

The power of random effects meta-analysis: supplementary materials

Dan Jackson and Rebecca Turner

September 15, 2016

This web appendix contains two Appendices that prove some necessary results in the main paper.

Appendix one

In this Appendix we derive the power of studies that contribute to a random-effects meta-analysis. We require the result that, assuming $a > 0$,

$$\int_{-\infty}^{\infty} \Phi((x-b)/a)\phi(x)\mathrm{d}x = \Phi\left(\frac{-b}{\sqrt{1+a^2}}\right) \tag{1}$$

where $\phi(\cdot)$ is the standard normal probability density function. This is most easily shown by considering the joint distribution of two independent standard normal variables X and Y, and evaluating $P(Y \leq (X-b)/a)$ using a double integral. Evaluating the inner integral immediately yields the left hand side of (1). Then, because $a > 0$, another way to more directly evaluate this probability is $P(Y \leq (X-b)/a) = P(aY - X + b \leq 0)$; $aY - X + b \sim N(b, 1+a^2)$, which yields the right hand side of (1).

From the main paper we have that the study specific powers are $\beta_i(\delta_i, \sigma_i) = 1 + \Phi(-Z_a + \delta_i/\sigma_i) - \Phi(Z_a + \delta_i/\sigma_i)$ and under the random effects model we have $\delta_i \sim N(\delta, \tau^2)$. Hence the power of the ith study in terms of δ is obtained by integrating out the random effects

$$\beta(\delta, \tau^2, \sigma_i) = \int_{-\infty}^{\infty} (1 + \Phi(-Z_a + x/\sigma_i) - \Phi(Z_a + x/\sigma_i))\frac{1}{\tau}\phi((x-\delta)/\tau)\mathrm{d}x \tag{2}$$

Using (1) the integral in (2) can be evaluated, after changing variables in the integration, as

$$\beta(\delta, \tau^2, \sigma_i) = 1 + \Phi\left((-Z_a\sigma_i + \delta)/\sqrt{\sigma_i^2+\tau^2}\right) - \Phi\left((Z_a\sigma_i + \delta)/\sqrt{\sigma_i^2+\tau^2}\right) \tag{3}$$

Assuming that the distributions of σ_i and δ_i are independent, and approximating the distribution of σ_i with its empirical distribution, upon taking the sample average of powers in (3) we obtain equation (5) of the main paper.

Another more direct way to obtain (3) is to recognise that we have evaluated the expectation of $\beta_i(\delta_i, \sigma_i)$ over the distribution of δ_i. $\beta_i(\delta_i, \sigma_i)$ is the probability of rejecting the study specific null hypothesis given δ_i (and σ_i). From the law of total expectation, this expectation is the (unconditional on δ_i) probability of rejecting the study specific null hypothesis, so that we can also calculate (3) as the probability that $Y_i \sim N(\mu, \sigma_i^2 + \tau^2)$ lies further than $Z_a \sigma_i$ away from μ_0.

Appendix two

In this Appendix we derive the distribution of the test statistic for the special case where all studies are the same 'size' ($\sigma_i^2 = \sigma^2$) for all i. This extends previous work that derives this distribution under the assumption that $\mu_0 = \mu$, so that $\delta = 0$ (Jackson and Bowden 2009). For this special case the DerSimonian and Laird, REML and Paule-Mandel estimators become equivalent and, before truncation, we have $\sigma^2 + \hat{\tau}^2 = s^2$, where s^2 is the sample variance. This means that $\hat{\tau}^2 = \max(0, s^2 - \sigma^2)$ for all three estimators. This result is easily established for the DerSimonian and Laird and Paule-Mandel estimators from equating $\sigma_i^2 = \sigma^2$ in their estimating equations and a little algebra, where we also note that the pooled estimators of μ that appear in the estimating equations are equal to $\bar{y}$. For REML this result is similarly easily obtained from the expression for the restricted log likelihood given by Normand (1999), her page 336, upon setting $\sigma_i^2 = \sigma^2$, differentiating with respect to τ^2 and setting the resulting expression to zero. The data are independent and identically distributed normal random variables when all studies are the same size, so that standard textbook results apply; in particular the sample mean $\bar{y}$ and the sample variance s^2 follow well known distributions and are independent.

Let E denote an indicator random variable where $E = 0$ if $\hat{\tau}^2 = 0$ and $E = 1$ if $\hat{\tau}^2 > 0$. We then evaluate the cumulative distribution function of the test statistic T, $P(T \leq t)$, as

$$P(T \leq t) = P(E = 0)P(T \leq t | E = 0) + \{1 - P(E = 0)\} P(T \leq t | E = 1) \quad (4)$$

and we consider the special case where $\sigma_i^2 = \sigma^2$ for all i. From the the result that $\hat{\tau}^2 = \max(0, s^2 - \sigma^2)$, $E = 1$ is equivalent to $s^2 > \sigma^2$ and $E = 0$ is

equivalent to $s^2 \le \sigma^2$. Jackson and Bowden (2009) show that

$$P(E=0) = P(s^2 \le \sigma^2) = \Gamma_1\left(\frac{k-1}{2}, \frac{(1-I^2)(k-1)}{2}\right) \tag{5}$$

Given that $E = 0$ the test statistic becomes $T = (\bar{y} - \mu_0)/\sqrt{\sigma^2/k}$. Since $\bar{y}$ and s^2 are independent, and the event that $E = 0$ is equivalent to $s^2 \le \sigma^2$, we have that the unconditional distribution of T and its corresponding conditional distribution given that $E = 0$ are the same and are both $T \sim N(\Delta, 1/(1-I^2))$, where $I^2 = \tau^2/(\sigma^2+\tau^2)$ and $\Delta = \delta\sqrt{k}/\sigma$. Hence

$$P(T \le t|E=0) = \Phi\left((t-\Delta)\sqrt{1-I^2}\right) \tag{6}$$

The most difficult term to derive is is $P(T \le t|E = 1)$. Given that $E = 1$ the test statistic T becomes $T = (\bar{y} - \mu_0)/\sqrt{s^2/k}$. We write $Y = \sqrt{k}(\bar{y}-\mu_0)/\sqrt{\sigma^2+\tau^2} \sim N(\Delta\sqrt{1-I^2}, 1)$, $W = ((k-1)/(\sigma^2+\tau^2))s^2 \sim \chi^2_{k-1}$ and $X = \sqrt{W/(k-1)}$, where X and Y are independent, so that

$$T|(E=1) = \frac{Y}{X}|\left(X > \sqrt{1-I^2}\right)$$

because the event that $E = 1$ is equivalent to $s^2 > \sigma^2$ and so is also equivalent to $X > \sqrt{1-I^2}$. Starting with the χ^2 probability density function of W and changing variables gives the probability density function of X as $f(x) = 2(k-1)x\chi^2_{k-1}((k-1)x^2)$ where $\chi^2_{k-1}(\cdot)$ is the probability density function of a χ^2 distribution with $(k-1)$ degrees of freedom. Since the event that $T \le t$ is equivalent to $Y \le tX$ we can evaluate

$$\{1-P(E=0)\}P(T \le t|E=1) = 2(k-1)\int_{\sqrt{1-I^2}}^{\infty} x\chi^2_{k-1}((k-1)x^2)\int_{-\infty}^{tx}\phi\left(y-\Delta\sqrt{1-I^2}\right)\mathrm{d}y\mathrm{d}x$$

so that

$$\{1-P(E=0)\}P(T \le t|E=1) = 2(k-1)\int_{\sqrt{1-I^2}}^{\infty} x\Phi\left(tx-\Delta\sqrt{1-I^2}\right)\chi^2_{k-1}\left((k-1)x^2\right)\mathrm{d}x \tag{7}$$

where we have obtained the probability that $Y \le tX$ and $X > \sqrt{1-I^2}$ by integrating over the region where both criteria are satisfied. Substituting (5), (6) and (7) into (4) gives equation (9) of the main paper.
